# Supplementary material for: Anti-Staphylococcal, Anti-Candida, and Free-Radical Scavenging Potential of Soil Fungal Metabolites: A Study Supported by Phenolic Characterization and Molecular Docking Analysis
Source: Curr Issues Mol Biol. 2023 Dec 28;46(1):221–43. doi: 10.3390/cimb46010016 (PMC10814734; doi:10.3390/cimb46010016)
Supplement: Supplementary file 1 [file cimb-46-00016-s001.zip › cimb-2782831-supplementary.pdf]

Contents

Figure	Page
**Figure S1.** HPLC chromatogram of phenolic components in EtOAc extract from *T. harzianum* AUMC 15440.	3
**Figure S2.** HPLC chromatogram of phenolic components in EtOAc extract from *T. harzianum* AUMC 15443.	3
**Figure S3.** HPLC chromatogram of phenolic components in EtOAc extract from *A. aureolatus* AUMC 15441.	4
**Figure S4.** HPLC chromatogram of phenolic components in EtOAc extract from *A. aureolatus* AUMC 15446.	4
**Figure S5.** HPLC chromatogram of phenolic components in EtOAc extract from *T. nidulans* AUMC 15444.	5
**Figure S6.** HPLC chromatogram of phenolic components in EtOAc extract from *A. terreus* AUMC 15447**.**	5
**Figure S7.** HPLC chromatogram of phenolic components in EtOAc extract from *A. terreus* AUMC 15448**.**	6
**Figure S8.** HPLC chromatogram of phenolic components in EtOAc extract from *P. crustosum* AUMC 15445.	6
**Figure S9.** HPLC chromatogram of phenolic components in EtOAc extract from *P. novae-zeelandiae* AUMC 15442**.**	7

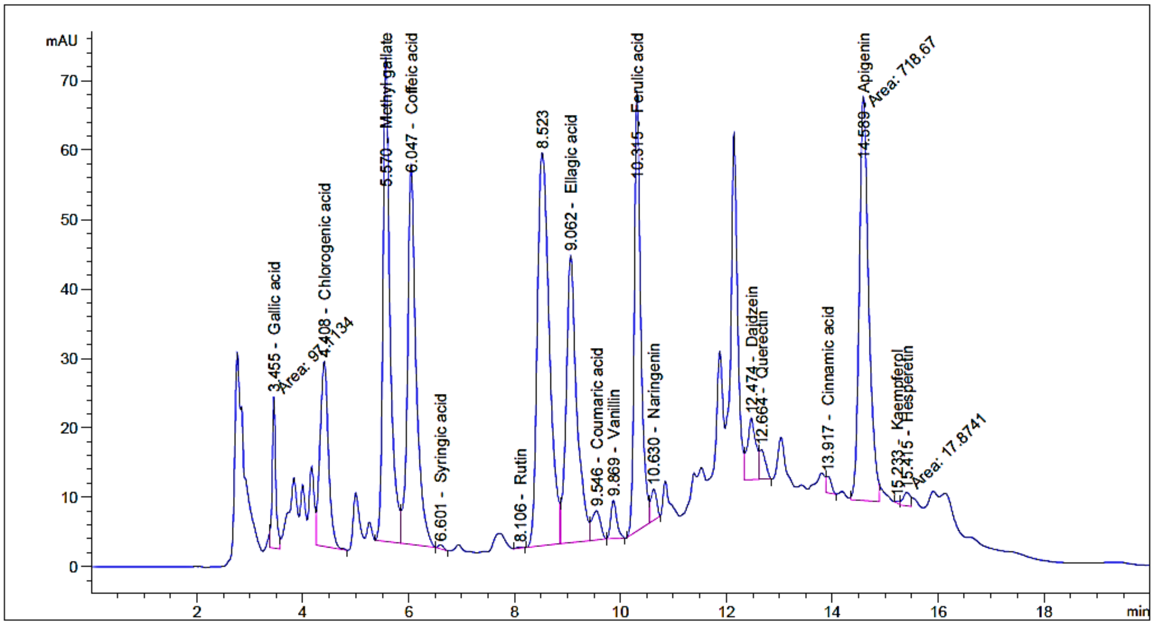

Figure S1. HPLC chromatogram of phenolic components in EtOAc extract from *T. harzianum* AUMC 15440.

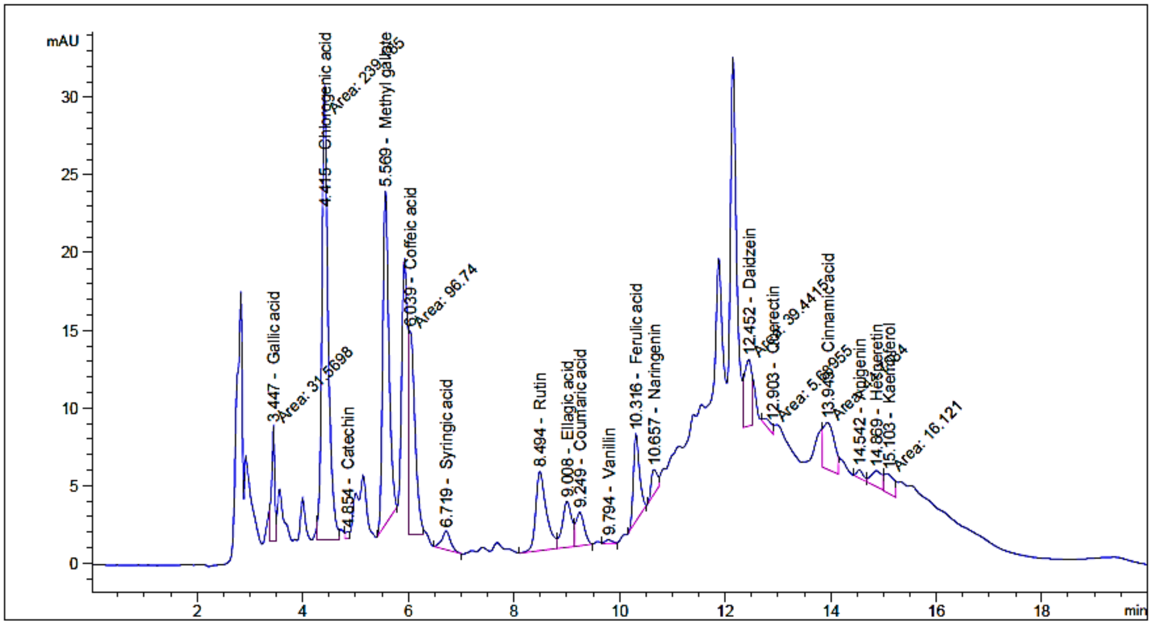

Figure S2. HPLC chromatogram of phenolic components in EtOAc extract from *T. harzianum* AUMC 15443**.**

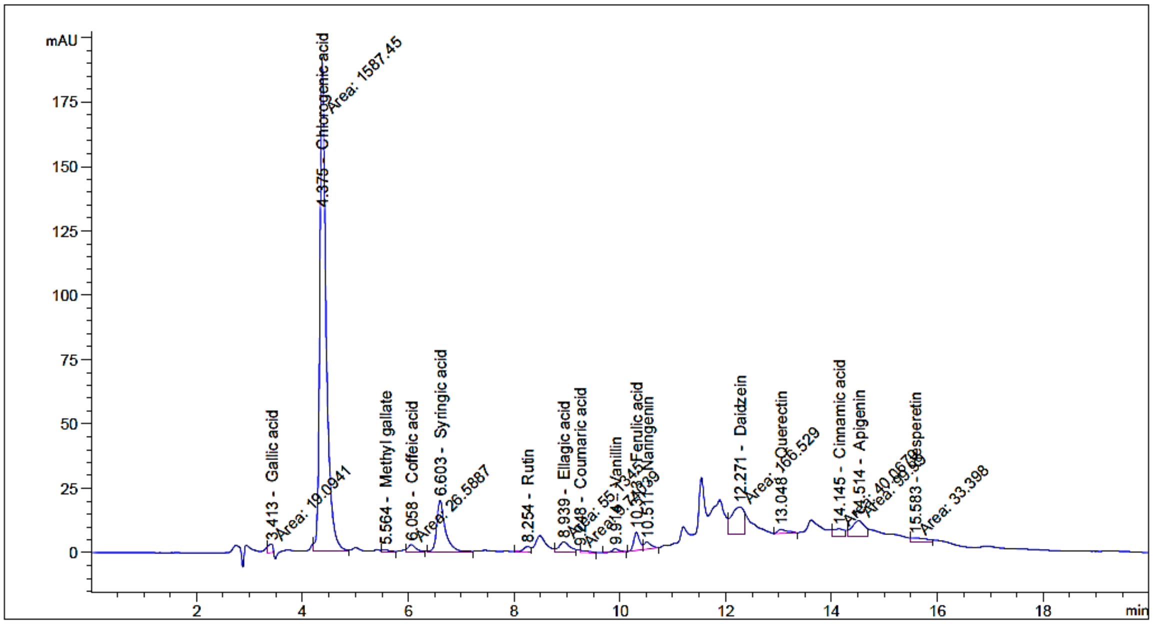

Figure S3. HPLC chromatogram of phenolic components in EtOAc extract from *A. aureolatus* AUMC 15441.

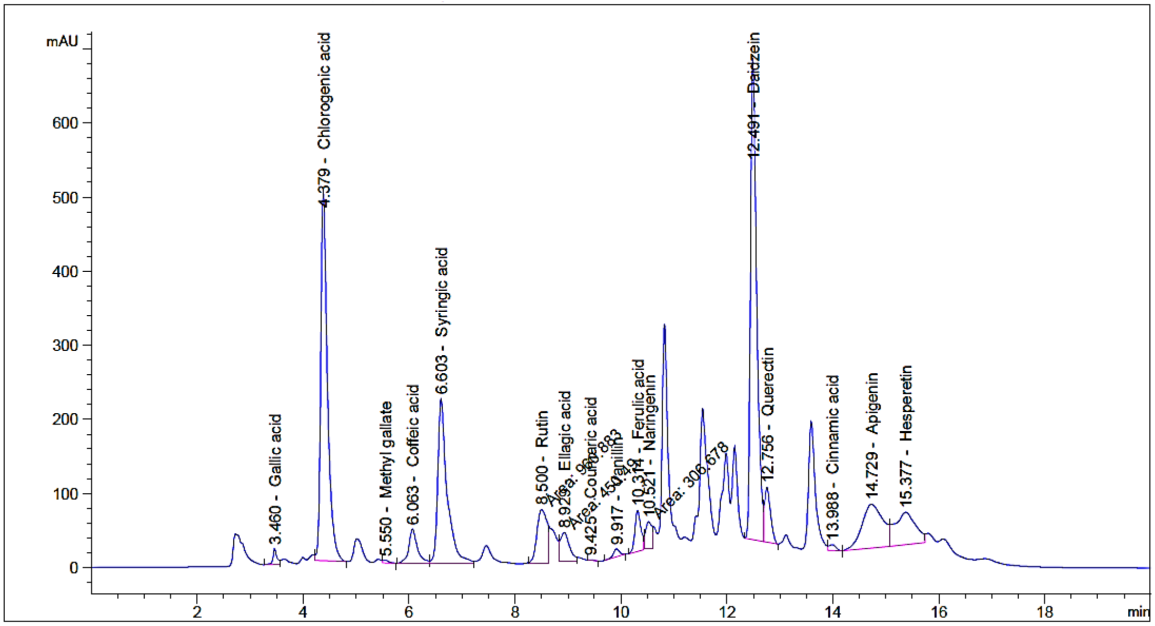

Figure S4. HPLC chromatogram of phenolic components in EtOAc extract from *A. aureolatus* AUMC 15446.

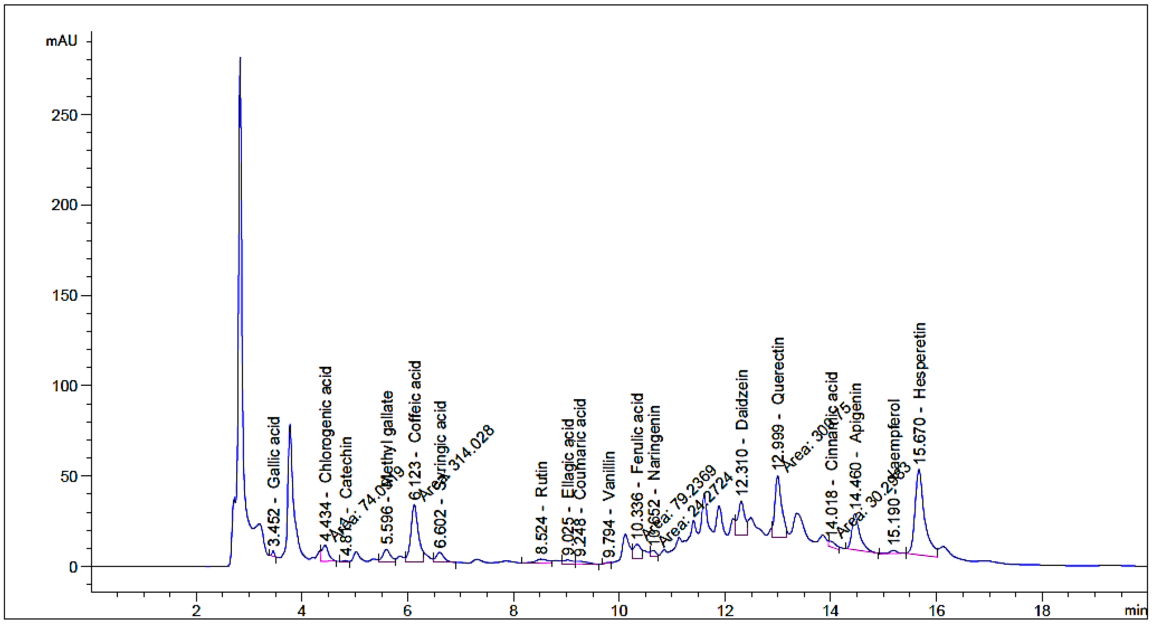

Figure S5. HPLC chromatogram of phenolic components in EtOAc extract from *T. nidulans* AUMC 15444.

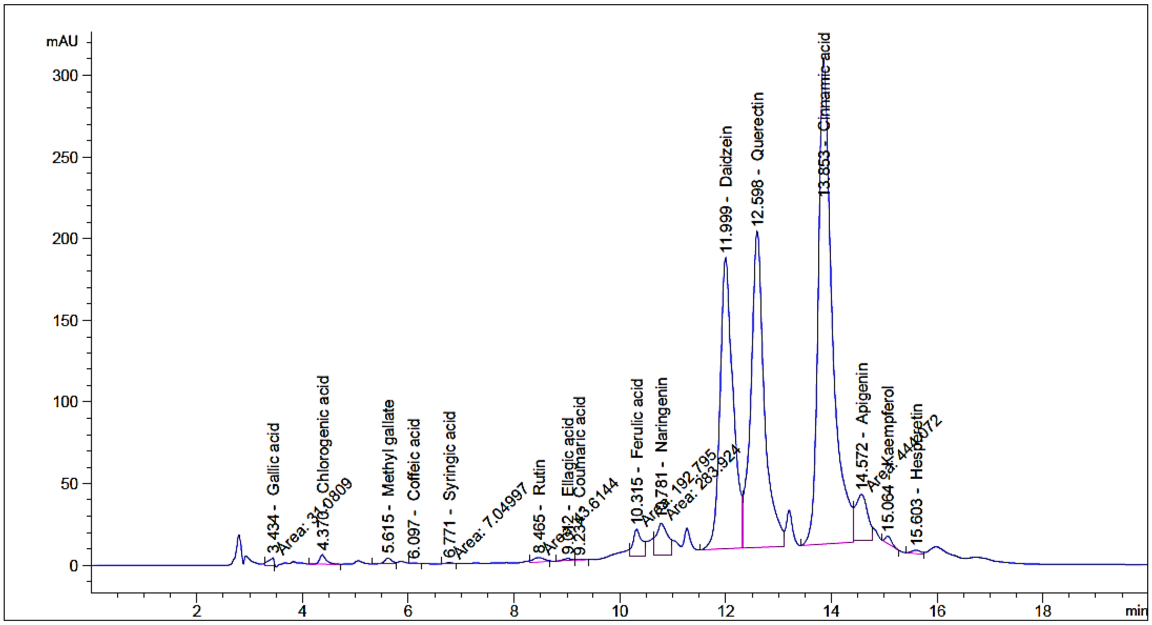

Figure S6. HPLC chromatogram of phenolic components in EtOAc extract from *A. terreus* AUMC 15447.

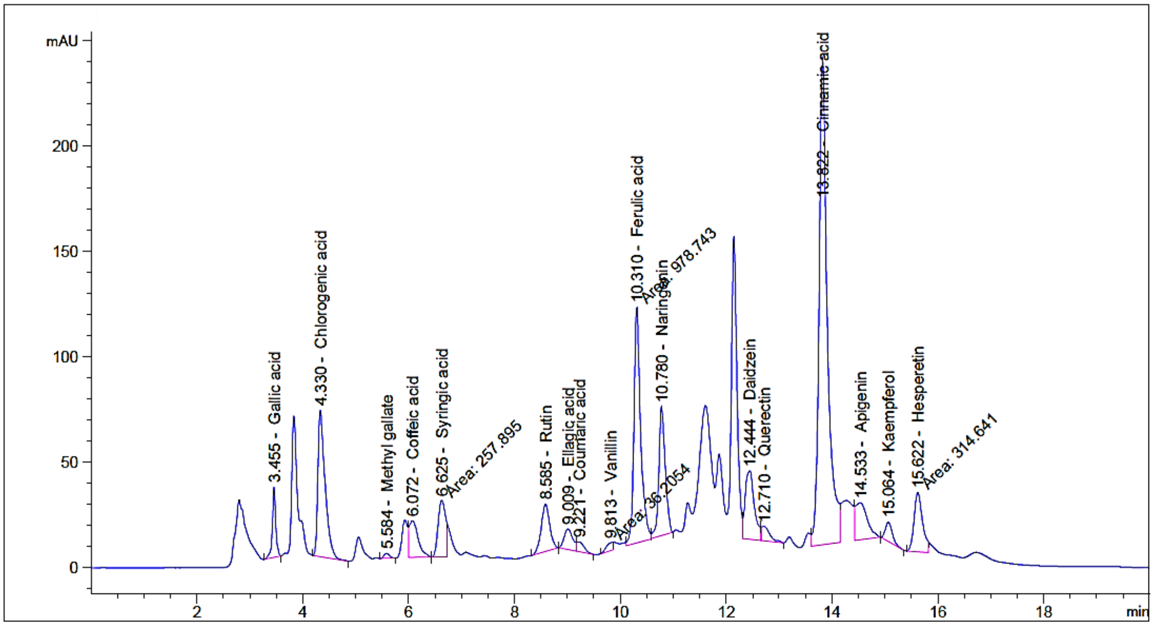

Figure S7. HPLC chromatogram of phenolic components in EtOAc extract from *A. terreus* AUMC 15448**.**

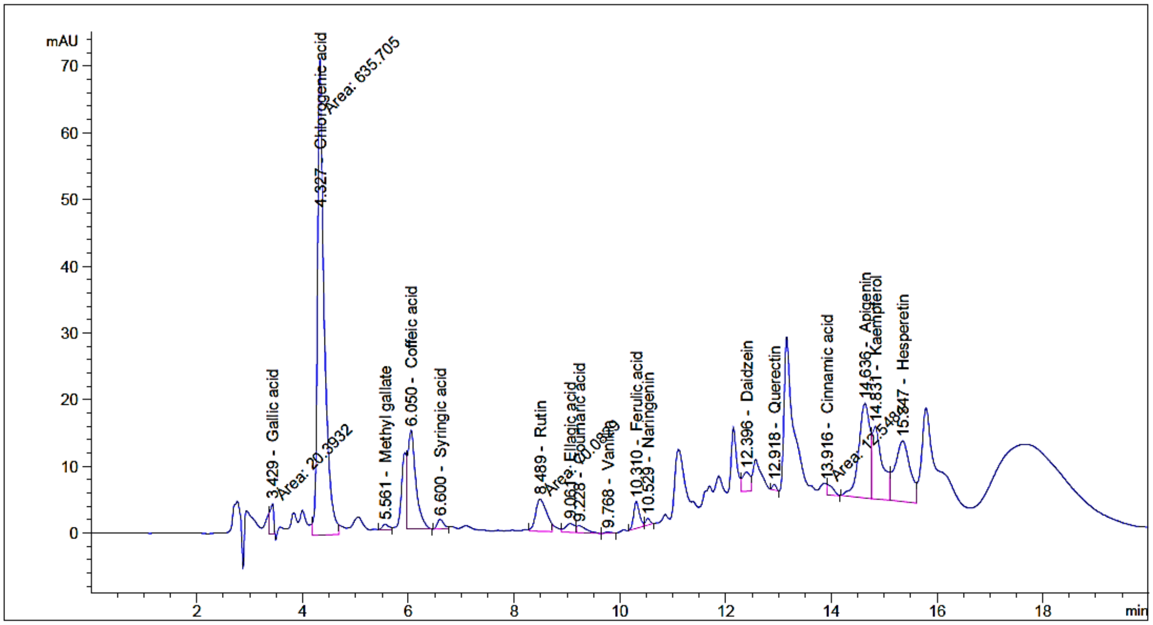

Figure S8. HPLC chromatogram of phenolic components in EtOAc extract from *P. crustosum* AUMC 15445.

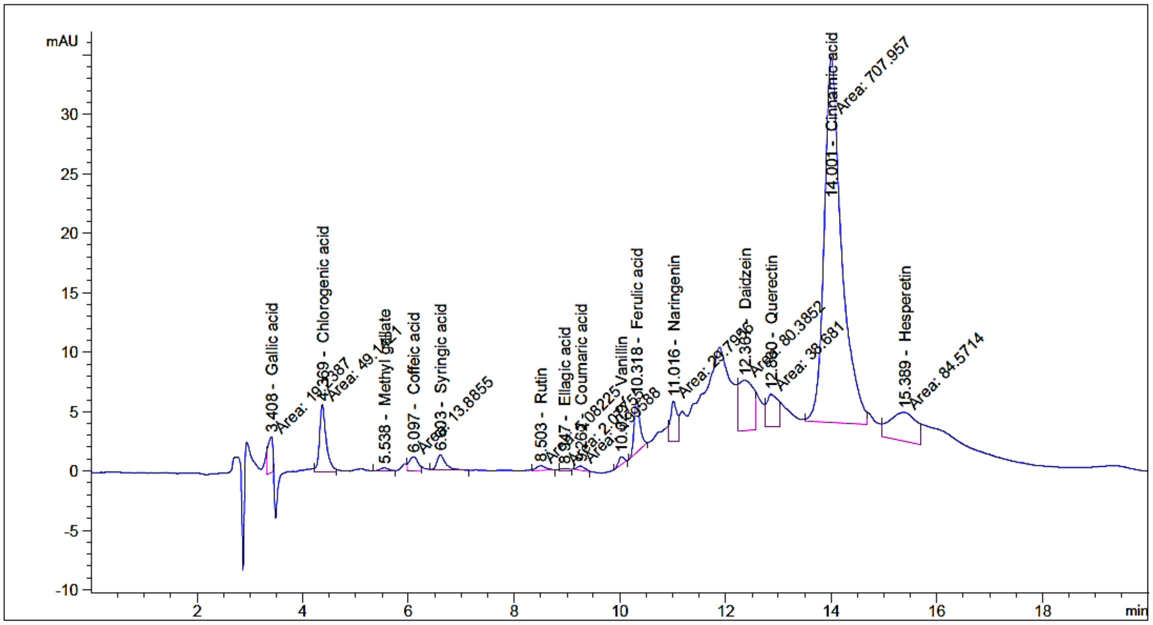

Figure S9. HPLC chromatogram of phenolic components in EtOAc extract from *P. novae-zeelandiae* AUMC 15442**.**
